# Clinical and radiological instability following standard fenestration discectomy

**DOI:** 10.4103/0019-5413.55465

**Published:** 2009

**Authors:** Amrithlal A Mascarenhas, Issac Thomas, Gaurav Sharma, Joe Joseph Cherian

**Affiliations:** Department of Orthopaedics, St Johns National Academy of Health Sciences, Bangalore, India

**Keywords:** Discectomy, fenestration, instability

## Abstract

**Background:**

Post-surgical lumbar instability is an established complication but there is limited evidence in the literature regarding the incidence of lumbar instability following fenestration and discectomy. We analyzed our results following fenestration discectomy with a special focus on instability.

**Materials and Methods:**

Eighty-three patients between the age of 17 and 52 years who had undergone fenestration discectomy for a single-level lumbar intervertebral disc prolapse were followed-up for a period of 1–5 years. The criteria for instability included “instability catch,”, “painful catch,” and “apprehension.” The working capacity of the patient and the outcome score of the surgery were assessed by means of the Oswestry disability score and the Prolo economic and functional outcome score. Flexion-extension lateral radiographs were taken and analyzed for abnormal tilt and translation.

**Results:**

Of the 83 patients included, 70 were men and 13 were women, with an average age of 37.35 years (17–52 years) at 5 years follow-up. Clinical instability was seen in 10 (12.04%) patients. Radiological instability was noted in 29 (34.9%) patients. Only six (60%) of the 10 patients who demonstrated clinical instability had radiological evidence of instability. Twenty (68.96%) patients with radiological instability were asymptomatic. Three (10.34%) patients with only radiological instability had unsatisfactory outcome. The Oswestry scoring showed an average score of 19.8%. Mild disability was noted in 59 (71.08%) patients and moderate disability was seen in 24 (28.91%) patients. None of the patients had severe disability. These outcomes were compared with the outcomes in other studies in the literature for microdiscectomy and the results were found to be comparable.

**Conclusion:**

The favorable outcome of this study is in good agreement with other studies on microdiscectomy. Clinical instability in 12.04% of the patients is in agreement with other studies. Radiological signs of instability are seen even in asymptomatic patients and so are not as reliable as clinical signs of instability. Standard fenestration discectomy does not destabilize the spine more than microdiscectomy.

## INTRODUCTION

Segmental instability of the lumbar spine is regarded as one of the sources of failed back surgery syndrome.^1^–^4^ Spinal instability is abnormal motion between two or more vertebrae.^5^,^6^ It is defined as the loss of ability of the spine under physiological loads to maintain its patterns of displacements so there are no initial or additional neurological deficits, no major deformity and no incapacitating pain.^7^ Extensive movement may cause mechanical deformation of the intraspinal nerve tissue and thereby induce pain and/or neurological deficits.^8^ But, even a minor instability may cause irritation of the receptors related to facet joints or other components of the motion segment, resulting in local pain and/or reflexly painful muscle spasm.^8^ Repeated transgression will damage the stabilizing structures beyond physiological repair thus putting abnormal demands on secondary restraints. Wide laminectomy tends to result in lumbar spinal instability.^9^ A wide fenestration procedure is preferred to prevent the occurrence of post-operative instability.^10^

The aim herein is to study the incidence of lumbar instability at a spinal segment following fenestration discectomy and to study the correlation between the clinical signs, symptoms, and radiological instability with the outcome.

## MATERIALS AND METHODS

**Figure 1:**
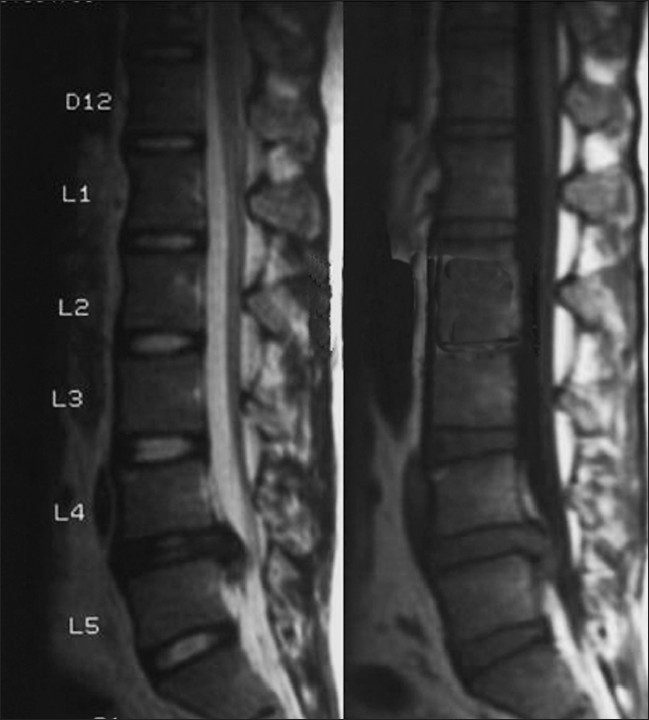
Sagittal section (T1 and T2 weighted) MRI showing herniated disc at L4-5 level

**Figure 2:**
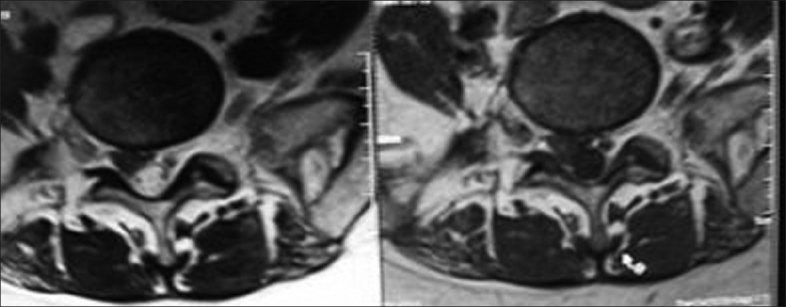
Axial sections of MRI lumbar spine showing right sided disc herniation compressing the traversing nerve root

This is a retrospective study with a prospective follow-up, which included 83 patients who were between 17 and 52 years and who had undergone fenestration discectomy for a single-level lumbar intervertebral disc prolapse, being followed-up for a period of 1–5 years (between 2002 and 2007). Patients with single-level disc herniation and planned for fenestration discectomy, having no clinical or radiological instability before surgery were included in the study [Figures 1 and 2].

Patients who underwent more than one level of fenestration discectomy and had clinical or radiological instability were excluded from the study. Also, patients with features of lumbar canal stenosis were excluded from the study.

### Operative procedure

Standard lumbar discectomy was carried out by the procedure described by Semmes^11^ and modified by Fager.^12^ The patient was given a general endotracheal anesthesia and placed on the operating table in the knee-chest position. A lateral lumbar radiograph then confirmed the correct interspace, which was then marked. A midline incision followed by a unilateral subperiosteal dissection of the muscles and tendons from the spinous processes and laminae was performed. Bilateral dissection was carried out in case of bilateral root compression. The ligamentum flavum was excised till the neural tube and a small laminotomy was carried out to expose the outer part of the nerve root compressed by the disc fragment. Once the root was retracted, the bulging annulus was identified and incised in a cruciate fashion, and discectomy was performed. The target root and interspace were explored to ensure complete decompression of the thecal sac and root sleeve. The wound was then closed in three layers. The operation lasted for 1 h.^13^ Post-operatively, the patients were encouraged to walk independently on the day after surgery. Back strengthening exercises were started as and when pain permitted. Back care and posture correction were taught. Lifting weights was permitted at 3 months post-surgery.

On follow-up, a detailed history and clinical examination was carried out. Patients were regularly followed-up at yearly intervals of 1, 2, 3, 4, and 5 years. Patients were assessed for lumbar spinal instability.^14^–^19^ The clinical criteria included “instability catch,” “painful catch,” and “apprehension.” Instability catch was considered when the patient experienced a sudden attack of low-back pain while returning to an erect posture from a bent position. Painful catch was considered when the patient was asked to lift up his leg and let it go slowly down to the table but was unable to do so, resulting in a sudden drop of the leg due to a sharp pain in the low-back region. Apprehension was considered as being present if the patient experienced anxiety resulting from a sudden sense of collapse of the low back because of sudden onset of back pain while moving.

The working capacity of the patient and the outcome score of surgery were assessed by means of the Oswestry disability score and the Prolo economic and functional outcome score.

**Figure 3:**
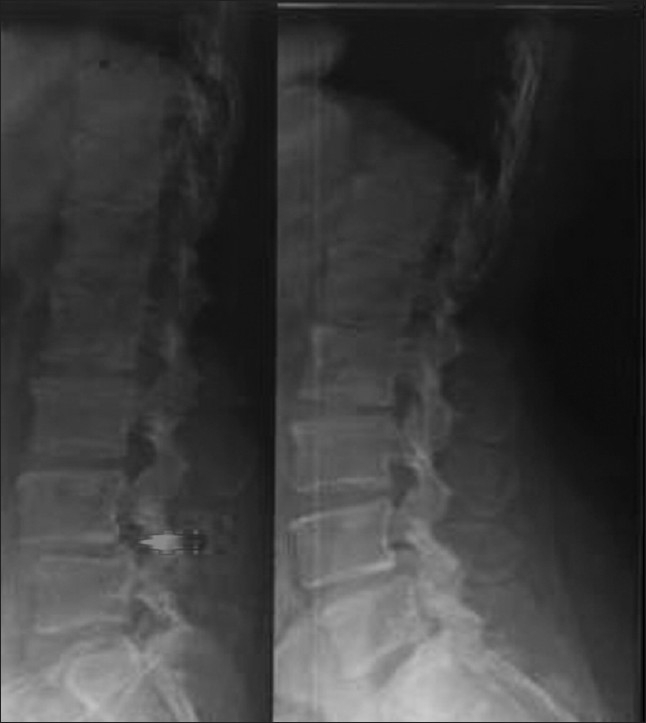
Dynamic flexion/extension views of the lumbar spine one year post-surgery showing translational as well as angular instability at L4- 5 level

Flexion-extension lateral radiographs were taken and analyzed for abnormal tilt and translation [Figure 3]. Abnormal sagittal tilt of more than 15^°^ and abnormal translation of more than 8% of the upper end plate of the inferior vertebra were taken as positive.^18^,^20^,^21^

All surgeries were performed following a strict protocol for fenestration discectomy by the authors and the 83 patients were reviewed and examined by a single author (First author).

## RESULTS

Of the 83 patients who were followed-up, there were 70 men and 13 women, with an average age of 37.35 years (17–52 years). Operated levels were as follows: L4-5 (n=54), L5-S1 (n=24), and L3-4 (n=5) in number. The mean duration of follow-up was 3.3 years (range 1.2–5 years), wherein 24 patients were followed-up for 5 years, 52 patients for 3 years, and the remaining for less than 3 years.

Clinical instability as per the criteria noted was seen in 10 (12.04%) patients, all of whom had demonstrated instability catch. The other signs were absent. Nineteen (22.89%) of our patients had complaints of back pain on and off.

Radiological instability was noted in 29 (34.9%) patients, of whom 16 showed tilt and 13 showed sagittal displacements. None of the patients had both tilt and displacement.

Only six (60%) of the 10 patients who demonstrated clinical instability had radiological evidence of instability. Twenty (68.96%) patients with radiological instability were asymptomatic. Three (10.34%) patients with only radiological instability had unsatisfactory outcome in terms of Oswestry disability index and the Prolo score.

The clinical and radiological signs in these patients were noted from the first follow-up, with no change at the final follow-up.

The Oswestry scoring showed an average score of 19.8%. Mild disability was noted in 59 (71.08%) patients and moderate disability was seen in 24 (28.91%) patients. None of the patients had severe disability. The mean score of those patients having minimal disability was 15.3%. The mean score of those who had moderate disability was 30.8%.The mean score of those who had clinical instability was 29% and the mean score of those who had radiological instability was 20%. The mean score of those patients who had both clinical and radiological instability was found to be 28%. All 10 (100%) patients who had clinical instability had moderate disability. Only nine (31.03%) of the patients with radiological signs of instability had a moderate disability score while the other 20 (68.96%) patients with radiological instability had only minimal disability. Five (6.02%) patients had moderate disability in spite of the absence of any signs of clinical or radiological instability.

The Prolo economic and functional outcome scoring showed good outcome in 60 (72.28%) patients and moderate outcome in 23 (27.7%) patients. All 10 (100%) patients with clinical instability showed a moderate outcome. Only nine (31.03%) patients with radiological signs of instability showed a moderate outcome while the remaining 20 (69.97%) patients had a good outcome. Four (4.81%) patients showed moderate outcome in spite of the absence of clinical or radiological signs of instability. The results are found to be in agreement/ homogenous. Thus we did not need to include any statistical analysis.

## DISCUSSION

In this study, the outcome of patients who underwent fenestration discectomy for lumbar disc herniation with special reference to post-operative instability of the lumbar spine was analyzed. Our results are in good agreement with similar studies performed after microdiscectomy.^8^,^22^

Lumbar instability can be verified both clinically and radiologically. The symptom of “instability catch” was found to be positive most commonly and was confirmed to be most significantly associated with an unsatisfactory post-operative outcome. The other two signs of “painful catch” and “apprehension” were not observed.

The correlation between spinal instability and an unsatisfactory outcome of a patient is very clear and is found to be significantly associated with the loss of work capacity, occurrence of low back pain, and unsatisfactory outcome in daily activity. In our study, all 10 (100%) patients who had clinical instability had moderate disability. Hence, it is important to look for lumbar instability not only as a prognostic factor but also to stress on the importance of clinical examination in the evaluation of this condition.^18^,^19^

Knutsson^23^ was the first to suggest translatory motion in the antero-posterior direction during flexion-extension as an indicator of spinal instability. Similar observations have been made by others.^24^–^27^ Many authors^28^,^29^ reported that poor surgical results are mainly seen in cases where abnormal slide or anterior tilting of the vertebral body occurs and the existence of the instability may cause post-operative back pain. In this study, radiological instability was noted in 29 (34.9%) patients, of which only nine (31.03%) patients had a moderate disability score while the other 20 (68.96%) patients had only minimal disability. Also, the fact that 20 patients with radiological instability were asymptomatic goes to show that radiological instability is a poor predictor of outcome as well as clinical instability. Similar results have been noted in other studies too wherein the radiological evidence has not correlated with the final outcome or the clinical instability.^24^,^30^,–^32^

The functional outcome in our patients as assessed by the Oswestry score and the Prolo score was in good agreement with the findings in many earlier studies in disc surgery. The satisfactory overall outcome in 71.08% of our patients was in agreement with the satisfactory outcome in 75% of the patients in the Kotilainen study and satisfactory outcome in 75–96% of the patients in previous studies on microdiscectomy.^22^,^33^,^34^ 22.89% of our patients occasionally suffered from low-back ache, which is in accordance with the findings of Spangfort.^35^

Up to 20% of the cases have shown to have detectable lumbar instability as a result of lumbar disc herniation. As a result, it has been hypothesized that in those patients suffering from instability, sparing operative methods like microdiscectomy and even percutaneous nucleotomy might be preferred to standard surgery (fenestration discectomy) in the treatment of lumbar disc disease.^8^ But, the results of this study show that this hypothesis need not necessarily be true as our results are found to be homogenous with the results of various other studies on microdiscectomy.

The favorable outcome of this study is in good agreement with other studies on microdiscectomy. Clinical instability in 12.04% of our patients is in agreement with other studies. Radiological signs of instability are seen even in asymptomatic patients and so are not as reliable as clinical signs of instability.

## CONCLUSION

Standard fenestration discectomy does not destabilize the spine more than microdiscectomy. Further studies are required to truly evaluate the development and progression of segmental instability in patients treated for lumbar disc herniation using different surgical methods.
